# An Examination of the Neutralization of In Vitro Toxicity of Chinese Cobra (*Naja atra*) Venom by Different Antivenoms

**DOI:** 10.3390/biomedicines8100377

**Published:** 2020-09-25

**Authors:** Qing Liang, Tam Minh Huynh, Nicki Konstantakopoulos, Geoffrey K. Isbister, Wayne C. Hodgson

**Affiliations:** 1 Monash Venom Group, Department of Pharmacology, Biomedical Discovery Institute, Monash University, Clayton 3800, Australia; qing.liang@monash.edu (Q.L.); Tommy.Huynh@monash.edu (T.M.H.); Nicki.Konstantakopoulos@monash.edu (N.K.); geoff.isbister@gmail.com (G.K.I.); 2 Department of Emergency Medicine, The First Affiliated Hospital of Guangzhou Medical University, 151 Yanjiang Rd, Guangzhou 510120, China; 3 Clinical Toxicology Research Group, University of Newcastle, Callaghan 2308, Australia

**Keywords:** *Naja atra*, neurotoxicity, myotoxicity, venom, antivenom, snake

## Abstract

The Chinese Cobra (*Naja atra*) is an elapid snake of major medical importance in southern China. We describe the in vitro neurotoxic, myotoxic, and cytotoxic effects of *N. atra* venom, as well as examining the efficacy of three Chinese monovalent antivenoms (*N. atra* antivenom, *Gloydius brevicaudus* antivenom and *Deinagkistrodon acutus* antivenom) and an Australian polyvalent snake antivenom. In the chick biventer cervicis nerve-muscle preparation, *N. atra* venom (1–10 µg/mL) abolished indirect twitches in a concentration-dependent manner, as well as abolishing contractile responses to exogenous acetylcholine chloride (ACh) and carbamylcholine chloride (CCh), indicative of post-synaptic neurotoxicity. Contractile responses to potassium chloride (KCl) were also significantly inhibited by venom indicating myotoxicity. The prior addition of Chinese *N. atra* antivenom (0.75 U/mL) or Australian polyvalent snake antivenom (3 U/mL), markedly attenuated the neurotoxic actions of venom (3 µg/mL) and prevented the inhibition of contractile responses to ACh, CCh, and KCl. The addition of Chinese antivenom (0.75 U/mL) or Australian polyvalent antivenom (3 U/mL) at the t_90_ time point after the addition of venom (3 µg/mL), partially reversed the inhibition of twitches and significantly reversed the venom-induced inhibition of responses to ACh and CCh, but had no significant effect on the response to KCl. Venom (30 µg/mL) also abolished direct twitches in the chick biventer cervicis nerve-muscle preparation and caused a significant increase in baseline tension, further indicative of myotoxicity. *N. atra* antivenom (4 U/mL) prevented the myotoxic effects of venom (30 µg/mL). However, *G. brevicaudus* antivenom (24 U/mL), *D. acutus* antivenom (8 U/mL) and Australian polyvalent snake antivenom (33 U/mL) were unable to prevent venom (30 µg/mL) induced myotoxicity. In the L6 rat skeletal muscle myoblast cell line, *N. atra* venom caused concentration-dependent inhibition of cell viability, with a half maximal inhibitory concentration (IC_50_) of 2.8 ± 0.48 μg/mL. *N. atra* antivenom significantly attenuated the cytotoxic effect of the venom, whereas Australian polyvalent snake antivenom was less effective but still attenuated the cytotoxic effects at lower venom concentrations. Neither *G. brevicaudus* antivenom or *D. acutus* antivenom were able to prevent the cytotoxicity. This study indicates that Chinese *N. atra* monovalent antivenom is efficacious against the neurotoxic, myotoxic and cytotoxic effects of *N. atra* venom but the clinical effectiveness of the antivenom is likely to be diminished, even if given early after envenoming. The use of Chinese viper antivenoms (i.e., *G. brevicaudus* and *D. acutus* antivenoms) in cases of envenoming by the Chinese cobra is not supported by the results of the current study.

## 1. Introduction

There are approximately 205 species of snakes in China, of which more than 50 species are venomous [1]. The Chinese Cobra (*Naja atra*) is one of the top ten most venomous and clinically important species in China [2]. In China, *N. atra* is mainly distributed south of the Yangtze River, but is also found in Laos and Vietnam. Based on venomic data, Chinese *N. atra* venom contains a range of toxins, with cardiotoxins and short-chain neurotoxins being the most abundant components [3,4,5,6]. We have previously isolated a short-chain neurotoxin, α-Elapitoxin-Na1a, from Chinese *N. atra* venom [7]. However, it has been previously shown that short-chain neurotoxins dissociate readily from human nicotinic acetylcholine receptors (nAChRs) and are unlikely to contribute substantially to neurotoxicity in humans [7,8]. The major outcomes of envenoming by Chinese *N. atra* include severe wound necrosis or chronic necrotic ulceration for which large doses of antivenom are administered. Treatment also requires wound infection control and repeated surgical debridement, with the potential for the eventual amputation of limbs. However, marked neurotoxicity including respiratory muscle paralysis is relatively rare [2,9,10,11].

Antivenoms form the mainstay treatment of systemic snake envenoming. Currently available antivenoms in China include a monovalent *N. atra* antivenom, and a bivalent elapid (*N. atra* and *Bungarus multicinctus*) antivenom in Taiwan [2,10]. However, there are a lack of animal studies or clinical trials that demonstrate the efficacy of *N. atra* antivenom. Unfortunately, the use of non-specific antivenoms is common in mainland China given there are only monovalent snake antivenoms available, i.e., two for elapids: *N. atra* (Chinese Cobra) antivenom and *Bungarus multicinctus* (Chinese Krait) antivenom; and two for vipers: *Gloydius brevicaudus* (Short-Tailed Mamushi) antivenom and *Deinagkistrodon acutus* (Sharp-nosed Pit Viper) antivenom. *G. brevicaudus* or *D. acutus* antivenoms are advocated for the treatment of local necrosis in patients envenomed by *N. atra*, when specific antivenom is unavailable. However, there is no evidence for the cross-neutralizing ability of these antivenoms for myotoxicity or cytotoxicity, although patients envenomed by these vipers may also experience local necrosis in severe cases [2].

In this study, we examined the in vitro neurotoxic, myotoxic and cytotoxic effects of Chinese *N. atra* venom and evaluated the efficacy of Chinese *N. atra* monovalent antivenom in comparison to a polyvalent elapid antivenom (i.e., Australian polyvalent antivenom) and the possible protective effects of Chinese *G. brevicaudus* and *D. acutus* antivenoms against the myotoxicity and cytotoxicity induced by *N. atra* venom.

## 2. Experimental Section

### 2.1. Venom and Antivenoms

Freeze-dried *N. atra* venom was obtained from Orientoxin Co., Ltd. (Laiyang, Shandong, China). Chinese *N. atra* monovalent antivenom (Batch number: 20181202; expiry date: 27/12/2021), Chinese *G. brevicaudus* monovalent antivenom (Batch number: 20190605; expiry date: 18/06/2022), Chinese *D. acutus* monovalent antivenom (Batch number: 20190101; expiry date: 21/01/2022) were purchased from Shanghai Serum Biological Technology Co., Ltd. (Shanghai, China). Australian polyvalent snake antivenom (Batch number: 055517501; expiry date: 04/2013) was purchased from Seqirus (Melbourne, Australia). The amount of each antivenom required to neutralize in vitro neurotoxicity was based on the quantity of venom in the organ bath. While for the myotoxicity study, in order to achieve a sufficiently high concentration of antivenom for the venom, all antivenoms were tested at 40 µL/mL. According to the manufacturer’s instructions: 125 U of *N. atra* antivenom neutralizes 1 mg of *N. atra* venom; 1500 U of *G. brevicaudus* antivenom neutralizes 1–1.25 mg of *G. brevicaudus* venom; and 136 U of *D. acutus* antivenom neutralizes 1–3 mg of *D. acutus* venom. For the Australian polyvalent antivenom, 1 U of antivenom neutralizes 10 μg of venom from the species of snake against which the antivenom is raised (i.e., brown snake, death adder, mulga snake, taipan, tiger snake).

### 2.2. Chemicals and Reagents

The following chemicals and drugs were used: acetylcholine chloride (ACh; Sigma-Aldrich, St. Louis, MO, USA), carbamylcholine chloride (CCh; Sigma-Aldrich, St. Louis, MO, USA), d-tubocurarine chloride (d-TC; Sigma-Aldrich, St. Louis, MO, USA), potassium chloride (KCl, Ajax Finechem Pty. Ltd., Taren Point, Australia), bovine serum albumin (BSA; Sigma-Aldrich, St. Louis, MO, USA), 0.5% Trypsin-EDTA (Gibco Thermofisher, Melbourne, Australia), Penicillin/Streptomycin, Dulbecco’s Phosphate Buffered Saline, Dulbecco’s Modified Eagle Medium (DMEM) GlutaMAX TM, DMSO (Merck; Darmstach, Germany), CellTire 96 Aqueous One Solution Cell Proliferation Assay (MTS assay; Promega; Melbourne, Australia). All chemicals were dissolved or diluted in Milli-Q water unless otherwise stated.

### 2.3. Chick Biventer Cervicis Nerve-Muscle Preparation

Chickens (male; aged 4–10 days) were killed by exsanguination following CO_2_ inhalation. Two biventer cervicis nerve-muscle preparations were dissected from each chick and mounted in separate organ baths on wire tissue holders under 1 g resting tension. Preparations were maintained at 34 °C, bubbled with 95% O_2_ and 5% CO_2_, in 5 mL organ baths filled with physiological salt solution consisting of (in mM): 118.4 NaCl, 4.7 KCl,1.2 MgSO_4_, 1.2 KH_2_PO_4_, 2.5 CaCl_2_, 25 NaHCO_3_, and 11.1 glucose. Venom was dissolved in 0.05% (*w*/*v*) bovine serum albumin (BSA).

For neurotoxicity experiments, indirect twitches were evoked by stimulating the motor nerve at supramaximal voltage (0.1 Hz; 0.2 ms; 10–20 V) via an electronic stimulator. d-TC (10 μM) was then added to the preparations with the subsequent abolishment of twitches indicating that they were nerve-mediated. The twitches were then restored by washing the preparation with physiological salt solution. Electrical stimulation was stopped and contractile responses to exogenous ACh (1 mM for 30 s), CCh (20 μM for 60 s), and KCl (40 mM for 30 s) obtained. Electrical stimulation was then recommenced for at least 30 min before the addition of venom or antivenom. To examine the efficacy of antivenom to prevent venom-induced neurotoxicity, antivenom was added to the tissues 10 min before venom. To examine the efficacy of antivenom to reverse venom-induced neurotoxicity, antivenom was added to the tissues at the t_90_ time point (i.e., when the twitch height was inhibited by 90%). At the conclusion of each experiment, ACh, CCh, and KCl were re-added as above.

For myotoxicity experiments, the biventer cervicis muscle was directly stimulated (0.1 Hz; 2 ms) at supramaximal voltage (20–30 V). In these experiments the electrode was placed around the belly of the muscle and d-TC (10 μM) remained in the organ bath for the duration of the experiment. Venom was left in contact with the preparation until twitch blockade occurred, or for a maximum 3 h period. Venom was considered to be myotoxic if it inhibited twitches elicited by direct stimulation and/or caused a contracture of the skeletal muscle (i.e., increase in the baseline tension of the muscle). To examine the ability of antivenom to neutralize venom-induced myotoxicity (i.e., myotoxicity prevention study), tissues were equilibrated with antivenom for 10 min before venom was added.

Twitch responses and responses to exogenous agonists were measured via a Grass FT03 force displacement transducer and recorded on a PowerLab system (ADInstruments Pty Ltd., Bella Vista, Australia). Animal experiments were approved on 12 May 2017 by the Monash University Ethics Committee application MARP/2017/147. All experiments were performed in accordance with relevant guidelines and regulations.

### 2.4. Cell Culture Experiments

#### 2.4.1. Venom

Freeze-dried venom was reconstituted in distilled water on the day of use. Protein content was determined utilizing a BCA protein assay kit according to the manufacturer’s instructions. Briefly, venom (25 μL) was added in triplicate to a 96-well micro-titer plate. BSA solutions, diluted from 1–0.025 mg/mL, were used as reference standards and distilled water was used as the blank. Absorbance was measured at 562 nm utilizing VERSAmax tunable microplate reader (Molecular Devices, San Jose, CA, USA). Venom stock solutions were stored at 4 °C until required.

#### 2.4.2. Heat Inactivation of Fetal Bovine Serum (hiFBS)

Fetal bovine serum was heated to 56 °C for 30 min. Following heat-inactivation, serum was sterilized using a 0.22 μM Millipore filter (Sigma-Aldrch, North Ryde, Australia). Serum was dispensed into sterile centrifuge tubes and stored at −20 °C.

#### 2.4.3. Cells

The rat skeletal muscle myoblast cell line, L6, was purchased from The American Type Culture Collection (ATCC, Manassas, VA, USA). L6 cells were grown in 175 cm^2^ flasks (Nunc, Thermofisher, Melbourne, Australia) in culture media DMEM supplemented with 10% hiFBS and 1% penicillin/streptomycin (10% DMEM). Flasks were maintained at 37 °C with 5% CO_2_ and media was replenished every subsequent day. When the cells reached 80% confluence (assessed by eye using a light microscope), trypsin was then used to lift the cells. Cells were centrifuged and the cell pellet was re-suspended in culture media (35 mL). Cell suspension (100 μL/well) was aliquoted into four 96-well cell culture plates (92 wells/plate) (Nunc, Thermofisher, Melbourne, Australia). Plates were maintained at 37 °C in an atmosphere of 5% CO_2_. Media was replenished every second day until cells reached 90% confluence. For cell differentiation to occur (i.e., skeletal myoblast cells into skeletal myocytes), 10% DMEM was removed from wells and replaced with DMEM media supplemented with 2% hiFBS and 1% penicillin/streptomycin (2% DMEM). Plates were subsequently maintained at 37 °C in an atmosphere of 5% CO_2_. Media was replenished every second day, for one week, until cell differentiation (i.e., appearance of long striated cells, assessed by eye using a light microscope) was observed.

To maintain L6 stock, cells at passage 2 were lifted using trypsin and centrifuged. Supernatant was discarded and cell pellets were re-suspended in DMEM (20 mL) supplemented with 30% hiFBS and 10% DMSO. Cell suspension was aliquoted into individual 1 mL cryovials and stored in liquid nitrogen until required. Cells were passaged up to passage 12 before being discarded and a new vial of cells thawed.

#### 2.4.4. Cell Proliferation Assay (MTS Assay)

For cell viability experiments, media were removed from wells of differentiated L6 cell culture plates and the wells were washed once with pre-warmed PBS. Venom stock solution was diluted in 2% DMEM culture media to a final concentration of 100 μg/mL. This was subsequently serially diluted either 1.5-fold (100–0.016 μg/mL) or 1.3-fold (100–0.24 μg/mL). Dilutions (100 μL/well) were added in quadruplicate to wells in a cell culture plate. Culture media controls (i.e., cells and media with no venom) and media blanks (i.e., no cells) were also run in parallel. The plates were maintained at 37 °C with 5% CO_2_ for 24 h. Cell culture plates were subsequently removed from the incubator and washed with pre-warmed PBS three times. DMEM culture media (2%; 50 μL/well) and MTS solution (10 μL/well) were pre-mixed, and 60 μL added to each well. Plates were further incubated at 37 °C with 5% CO_2_ for 1 h. Absorbance was measured at 492 nm utilizing a VERSAmax tunable microplate reader (Molecular Devices, San Jose, CA, USA).

#### 2.4.5. Examining the Efficacy of Antivenom

Media was removed from wells of L6 cell culture plates and the wells were washed once with pre-warmed PBS. Venom stock solutions were diluted to a concentration of 0 (no venom), 2.5 (IC_50_ concentration range), 5 (twice IC_50_), 10 (initial concentration where 100% cell death occurs), or 30 μg/mL (concentration used in myotoxic study) in 2% DMEM culture media containing either no antivenom (venom only) or supplemented with *N. atra* monovalent antivenom (200 μL; 4 U/mL) Australian polyvalent snake antivenom (200 μL; 33 U/mL), *G. brevicaudus* antivenom (200 μL; 24 U/mL), or *D. acutus* antivenom (200 μL; 8 U/mL).

Each of the dilutions were added in triplicate to L6 culture plates and incubated at 37 °C with 5% CO_2_ for 24 h. Culture plates were removed from the incubator and washed with pre-warmed PBS three times. Fresh DMEM culture media (50 μL/well) and MTS solution (10 μL/well) were pre-mixed, and 60 μL/well was added to each well. The plates were further incubated at 37 °C with 5% CO_2_ for 1 h. Absorbance was measured at 492 nm utilizing VERSAmax tunable microplate reader (Molecular Devices, San Jose, CA, USA).

### 2.5. Data Analysis

For both in vitro neurotoxicity and myotoxicity experiments, twitch height in the chick biventer preparation was measured at regular time intervals and expressed as a percentage of the pre-venom twitch height. In neurotoxicity studies, the time taken for 90% inhibition of the twitch response (t_90_ value) was used to determine the potency of *N. atra* venom. Post-venom contractile responses to ACh, CCh, and KCl were expressed as a percentage of their original responses. In myotoxicity studies, the change in gram (g) of muscle baseline tension was measured every 10 min after venom addition. The maximum change in tension (g) and time (min) to achieve the maximum change in tension were also measured. Comparison of the effects of *N. atra* venom on twitch height, baseline tension, or time to reach maximum change in tension were made using a one-way analysis of variance (ANOVA). Comparison of responses to exogenous agonists before and after the addition of venom or vehicle was made using a Student’s paired *t*-test. All ANOVAs were followed by a Bonferroni’s multiple comparison post-hoc test. Data are presented as mean ± standard error of the mean (SEM) of n experiments. All data and statistical analyses were performed using PRISM 8.0.2 (GraphPad Software, San Diego, CA, USA, 2019).

For cell experiments, sigmoidal growth curves were graphed using Prism 8.0.2 as cell viability (% of maximum) versus log concentration of venom, and IC_50_ concentrations determined. Bar graphs displaying the efficacy of antivenoms were plotted as a percentage of cell viability. Cell viability was compared in the presence and absence of antivenom using a one-way ANOVA, with Bonferroni’s multiple comparisons test. For all statistical tests, *p* < 0.05 was considered statistically significant. Data are presented as mean ± standard error of the mean (SEM) of n experiments.

## 3. Results

### 3.1. In Vitro Neurotoxicity

#### 3.1.1. Concentration-Dependent Inhibition of Twitches and Exogenous Agonists Responses

**Figure 1:**
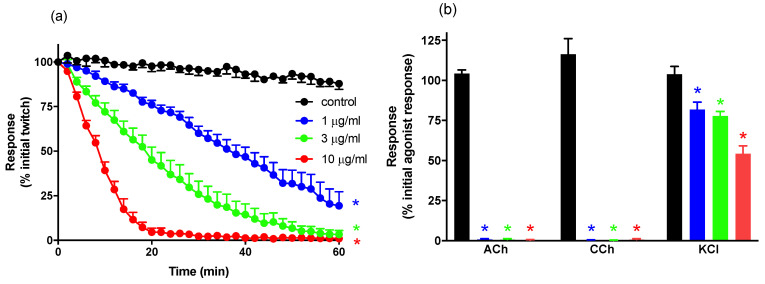
(**a**) The concentration-dependent neurotoxic effects of *N. atra* venom (1–10 µg/mL) on indirect twitches of the chick biventer cervicis nerve-muscle (CBCNM) preparation. (**b**) The concentration-dependent effects of *N. atra* venom (1–10 µg/mL) on contractile responses to acetylcholine chloride (ACh) (1 mM), carbachol (CCh) (20 µM), and potassium chloride (KCl) (40 mM) in the CBCNM. * *p* < 0.05, significantly different from (**a**) control at 60 min or (**b**) pre-venom response to same agonist. n = 6.

*N. atra* venom (1–10 µg/mL) caused concentration-dependent inhibition of indirect twitches of the chick biventer preparation, when compared to vehicle control (n = 6; one-way ANOVA, *p* < 0.05; Figure 1a). The potency of the neurotoxic effect of venom was determined by calculating t_90_ or t_50_ (i.e., if the twitch height to decrease by 90% or 50%, respectively) with values as follows: 1 µg/mL (t_50_ 36 ± 2 min), 3 µg/mL (t_90_ 43 ± 5 min), 10 µg/mL (t_90_ 17 ± 1 min). Venom (1–10 µg/mL) also abolished contractile responses to exogenous ACh (1 mM) and CCh (20 μM), indicating an action at the post-synaptic nerve terminal, and significantly inhibited responses to KCl (40 mM), indicative of myotoxicity (Figure 1b).

#### 3.1.2. In Vitro Neurotoxicity Antivenom Prevention Study

**Figure 2:**
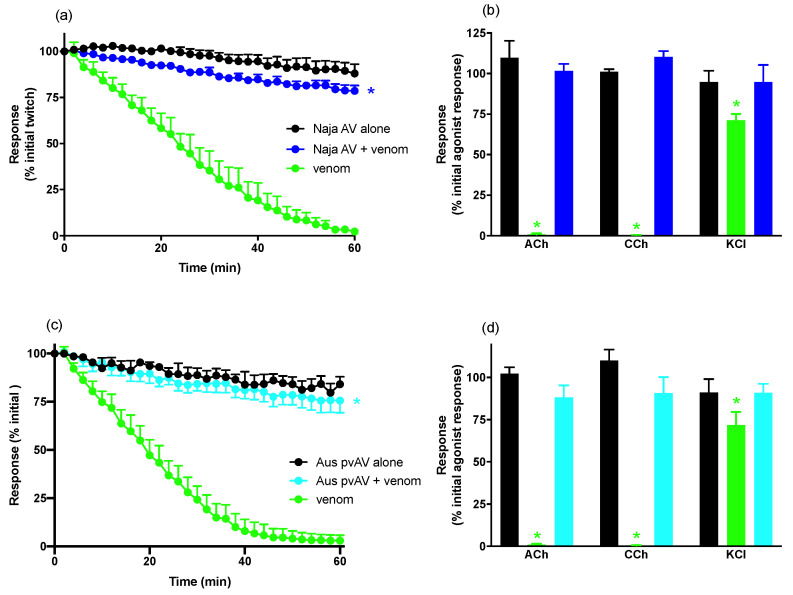
(**a**) The effects of *N. atra* venom (3 µg/mL) alone or with pre-addition of *Naja atra* antivenom (Naja AV; 0.75 U/mL) on indirect twitches of the CBCNM. (**b**) The effects of *N. atra* venom (3 µg/mL) alone or with pre-addition of Naja AV (0.75 U/mL) on contractile responses to ACh (1 mM), CCh (20 µM), and KCl (40 mM) in the CBCNM. (**c**) The effects of *N. atra* venom (3 µg/mL) alone or with pre-addition of Australian polyvalent antivenom (Aus pvAV; 3 U/mL) on indirect twitches of the CBCNM. (**d**) The effects of *N. atra* venom (3 µg/mL) alone or with pre-addition of Aus pvAV (3 U/mL) on contractile responses to ACh (1 mM), CCh (20 µM), and KCl (40 mM) in the CBCNM. * *p* < 0.05, significantly different compared to venom in the absence of antivenom at 60 min (**a**,**c**) or compared to pre-venom response to same agonist (**b**,**d**). n = 5–6.

The prior addition of Chinese *N. atra* monovalent antivenom (0.75 U/mL, 2× the recommended titre), or Australian polyvalent snake antivenom (3 U/mL, 10× the recommended titre), markedly attenuated the neurotoxic actions of venom (3 µg/mL) (Figure 2a,c) and prevented the inhibition of contractile responses to ACh, CCh, and KCl (Figure 2b,d).

#### 3.1.3. In Vitro Neurotoxicity Antivenom Reversal Study

**Figure 3:**
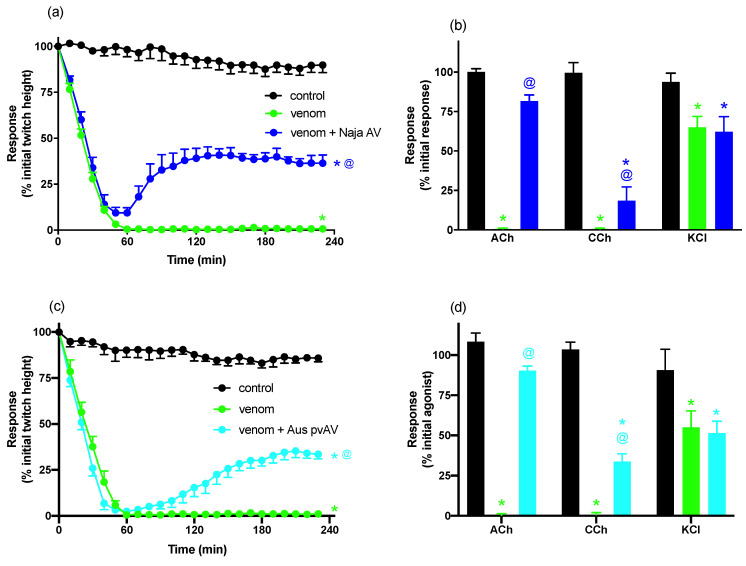
(**a**) The effects of *N. atra* venom (3 µg/mL) alone or with *Naja atra* antivenom (Naja AV; 0.75 U/mL) added at the t_90_ time point on indirect twitches of the CBCNM. (**b**) The effects of *N. atra* venom (3 µg/mL) alone or with Naja AV (0.75 U/mL) added at the t_90_ time point on contractile responses to ACh (1 mM), CCh (20 µM), and KCl (40 mM) in the CBCNM. (**c**) The effects of *N. atra* venom (3 µg/mL) alone or with Australian polyvalent antivenom (Aus pvAV; 3 U/mL) added at the t_90_ time point on indirect twitches of the CBCNM. (**d**) The effects of *N. atra* venom (3 µg/mL) alone or with Aus pvAV (3 U/mL) added at the t_90_ time point on contractile responses to ACh (1 mM), CCh (20 µM), and KCl (40 mM) in the CBCNM. * *p* < 0.05, significantly different compared to control at 230 min (**a**,**c**) or compared to pre-venom response to same agonist (**b**,**d**). ^@^ *p* < 0.05, significantly different compared to venom in the absence of antivenom at 230 min (**a**,**c**) or compared to response to agonist in the absence of antivenom (**b**,**d**), n = 5–6.

The addition of Chinese *N. atra* antivenom (0.75 U/mL, 2× the recommended titre), at the t_90_ time point, after the addition of *N. atra* venom (3 µg/mL), partially restored the twitch height, i.e., reaching 42 ± 5% (n = 6) of the initial pre-venom twitch height (Figure 3a). Chinese *N. atra* antivenom also significantly reversed the venom-induced inhibition of responses to ACh and CCh, while having no significant effect on the response to KCl (Figure 3b).

The addition of Australian polyvalent antivenom, (3 U/mL, 10× the recommended titre), at the t_90_ time point, after the addition of *N. atra* venom (3 µg/mL), partially restored the twitch height, i.e., reaching 35 ± 4% (n = 6) of the initial pre-venom twitch height (Figure 3c). The addition of Australian polyvalent antivenom also significantly reversed the inhibition of responses to ACh and CCh, while having no significant effect on the response to KCl (Figure 3d).

### 3.2. In Vitro Myotoxicity

**Figure 4:**
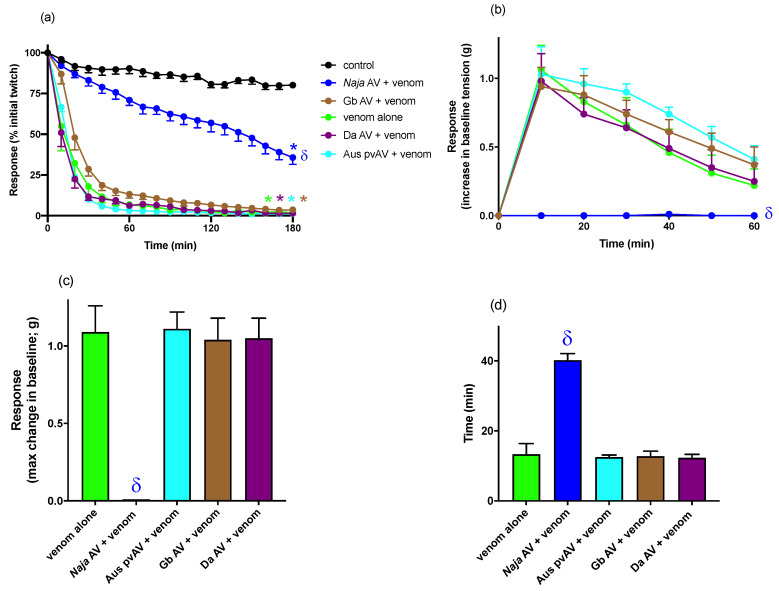
The myotoxic effects of *Naja atra* venom (30 µg/mL), in the presence and absence of different antivenoms, as indicated by the (**a**) change in twitch height in chick biventer cervicis preparation over 180 min; (**b**) change in baseline tension of the chick biventer cervicis preparation over 60 min; (**c**) max change in baseline gram tension achieved in 60 min; and (**d**) the time achieve max change in baseline tension. * *p* < 0.05, significantly different compared to control (**a**) at 180 min. ^δ^ *p* < 0.05, significantly different compared to venom alone at 180 min (**a**) or 60 min (**b**) or compared to venom in the absence of antivenom (**c**,**d**). n = 5–6. Antivenoms were added 10 min prior to venom. *N. atra* antivenom (*Naja* AV); *G. brevicaudus* antivenom AV (Gb AV); *D. acutus* antivenom AV (Da AV); Australian polyvalent AV (Aus pvAV).

*N. atra* venom (30 µg/mL) significantly inhibited twitches in the directly-stimulated chick biventer preparation, when compared to vehicle at 180 min (n = 5–6; one-way ANOVA, *p* < 0.05; Figure 4a).

The prior addition of Chinese *N. atra* monovalent antivenom 200 μL (4 U/mL, 1× the recommended titre) markedly attenuated, but did not prevent, twitch inhibition (n = 6; one-way ANOVA, *p* < 0.05; Figure 4a) and abolished the venom-induced increase in baseline tension compared to venom (30 µg/mL) alone (n = 5–6; one-way ANOVA, *p* < 0.05; Figure 4b–d), indicating partial attenuation of the myotoxic actions of *N. atra* venom.

In contrast, the prior addition of Australian polyvalent snake antivenom 200 μL (33 U/mL, 11× the recommended titre), *G. brevicaudus* antivenom 200 μL (24 U/mL, 0.5~0.7× the recommended titre) or Chinese *D. acutus* monovalent antivenom 200 μL (8 U/mL, 2~6× the recommended titre), failed to prevent or delay the venom-induced decrease in direct twitches(n = 5–6; Figure 4a) or venom-induced increase in baseline tension (n = 5–6; Figure 4b–d), indicating a lack of efficacy against the myotoxic actions of *N. atra* venom.

Control experiments (i.e., 200 µL of each antivenom alone) indicated the antivenoms had no direct effect on tissue viability over a period of 180 min (n = 5–6 for each antivenom).

### 3.3. Cell Viability Assay

#### 3.3.1. Venom Concentration–Response Curves

**Figure 5:**
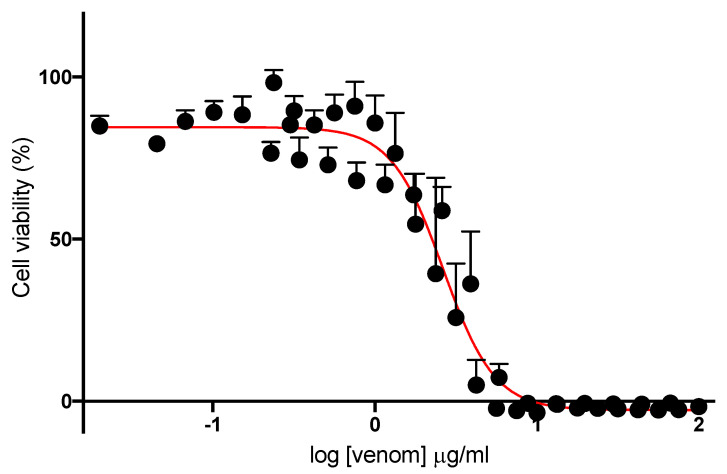
Concentration-dependent venom-induced inhibition of cell viability in L6 cells.

Treatment of L6 cells with *N. atra* venom resulted in a concentration-dependent inhibition of cell viability (Figure 5), with an IC_50_ of 2.8 ± 0.48 μg/mL.

#### 3.3.2. Cell-Based Proliferation Assay-Efficacy of Antivenoms

L6 cells were treated with 2% DMEM media supplemented with venom at concentrations of 0, 2.5, 5, 10, or 30 μg/mL and further supplemented with either no antivenom (i.e., venom alone) or with *N. atra* antivenom (200 μL; 4 U/mL), Australian polyvalent snake antivenom (200 μL; 33 U/mL), *G. brevicaudus* antivenom (200 μL; 24 U/mL), or *D. acutus* monovalent antivenom (200 μL; 8 U/mL).

**Figure 6:**
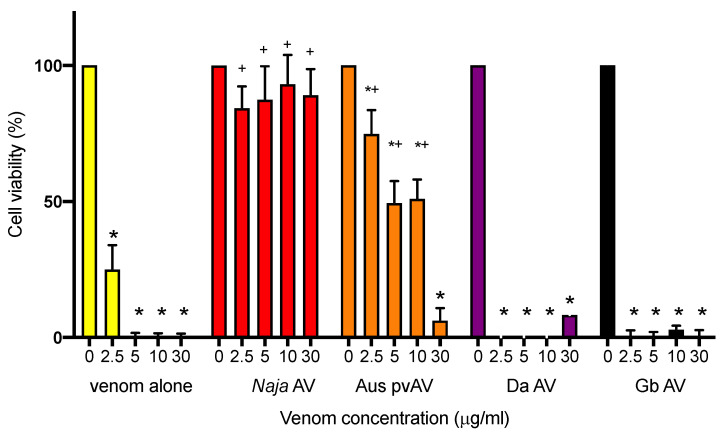
The effects of *N. atra* venom (0–30 μg/mL), in L6 cells, in the presence and absence of *N. atra* antivenom (*Naja* AV, 4 U/mL), *G. brevicaudus* antivenom AV (Gb AV, 24 U/mL), *D. acutus* antivenom AV (Da AV, 8 U/mL) or Australian polyvalent AV (Aus pvAV, 33 U/mL). * *p* < 0.05, significantly different from control (i.e., 0 venom); ^+^ *p* < 0.05, significantly different from same concentration of venom in the absence of antivenom.

*N. atra* venom caused a significant decrease in cell viability at all concentrations examined when compared to cells treated with media alone (*p* < 0.05; Figure 6). *N. atra* antivenom significantly attenuated the cytotoxic effect at all venom concentrations compared to control (*p* < 0.05; Figure 6). Australian polyvalent snake antivenom was less effective but still attenuated the cytotoxic effects at lower venom concentrations (i.e., 2.5–10 μg/mL; *p* < 0.05; Figure 6). Neither *G. brevicaudus* antivenom or *D. acutus* antivenom were able to prevent the cytotoxicity at any venom concentration examined (Figure 6).

## 4. Discussion

We have shown that *N. atra* venom from China displays potent in vitro neurotoxic, myotoxic and cytotoxic activity. The neurotoxic and cytotoxic effects of the venom were almost completely abolished by the prior addition of specific *N. atra* antivenom, whereas the myotoxic effects were only partially prevented. Interestingly, an Australian polyvalent antivenom, which is raised against the venoms from a range of Australian elapids (i.e., *Acanthophis antarcticus*, *Notechis scutatus*, *Oxyuranus scutellatus*, *Pseudechis australis*, and *Pseudonaja textilis*) and does not contain specific antibodies against *N. atra* venom, displayed similar activity against the neurotoxic effects of *N. atra* venom but was less effective against the cytotoxic effects and ineffective against the myotoxic effects. The Australian polyvalent antivenom was included in our study as the venoms of the Australian elapids contain a range of post-synaptic, pre-synaptic, and myotoxic components. These components are likely to have close structural similarities with some of the components in the venom of the Chinese cobra given we have previously shown that Australian Tiger snake (*N. scutatus*) antivenom prevents the in vitro neurotoxicity induced by *N. haje* (Egyptian cobra) venom [12], and Australian polyvalent snake antivenom prevents the in vitro neurotoxicity induced by *N. kaouthia* (monocled cobra) venom [13].

The two Chinese monovalent viper antivenoms (i.e., *G. brevicaudus* antivenom and *D. acutus* antivenom antivenom) had no efficacy against the myotoxic or cytotoxic effects of *N. atra* venom. We did not examine the efficacy of the Chinese viper antivenoms against the neurotoxic effects of *N. atra* venom as this is not a clinical outcome of envenoming by this species, and the antivenoms are used in China to treat the myotoxic symptoms of *N. atra* envenoming. Four monovalent snake antivenoms are available in mainland China, and cross-neutralization by using nonspecific antivenoms for snakebite is recommended in the Chinese 2018 Expert Consensus on snakebites [2]. However, it appears as though the two viper antivenoms have no efficacy against *N. atra* venom.

We used the chick biventer cervicis nerve–muscle preparation, which contains both focally- and multiply-innervated skeletal muscle fibers, to examine neurotoxicity and myotoxicity. This preparation enables the determination of the site of action of venoms/toxins, i.e., either at the pre-synaptic nerve terminal, post-synaptic nerve terminal or underlying skeletal muscle [14,15]. The time taken to cause 90% (i.e., t_90_) inhibition of nerve-mediated twitches can be used to compare the neurotoxic potency of venoms/toxins. *N. atra* venom abolished indirect twitches in a time-dependent and concentration-dependent manner, as well as inhibiting contractile responses to exogenous ACh and CCh, while reducing responses to KCl, indicating that it acts post-synaptically and has myotoxic effects on the tissue. The Chinese *N. atra* antivenom was highly efficacious when added prior to venom and was also able to partially reverse the inhibitory effects of the venom when added at the t_90_ time point. The failure to fully reverse the decline in twitch height is likely to be due to a number of factors including the contribution of myotoxins and the lack of reversibility of some neurotoxins. Indeed, we have previously shown that the short-chain post-synaptic neurotoxin α-Elapitoxin-Na1a, which accounts for approximately 9% of *N. atra* venom, displays pseudo-irreversible antagonism at the skeletal muscle nicotinic acetylcholine receptor and is only partially reversed by antivenom [7]. Interesting, the Australian polyvalent snake venom displayed similar efficacy against the neurotoxic effects of the venom indicating that the antigenic components in this antivenom, which is raised against a number of venoms from Australian elapid snakes containing postsynaptic and/or presynaptic neurotoxins, are able to recognize the neurotoxic components of *N. atra* venom.

Despite possible geographical differences in venom composition, the percentage of cardiotoxins and neurotoxins reported in *N. atra* venom ranges from 52–68% and 11–23%, respectively [3,4,6]. Cardiotoxins, which target cell membranes, are likely to be the main components contributing to the soft tissue necrosis and myotoxicity [16,17,18,19,20]. Indeed, *N. atra* venom has been shown to display high levels of cytotoxicity [21]. Although, as indicated above, *N. atra* venom is highly neurotoxic in vitro, it is only mildly neurotoxic in humans. This is most likely due to the neurotoxic components being short-chain neurotoxins which readily dissociate from human muscle nAChRs [7]. Interestingly, a recent study found that the post-synaptic α-neurotoxins in *N. atra* venom bind to the alpha-1 nAChR orthosteric site with selectivity towards the amphibian mimotope over lizard, avian and rodent mimotopes indicative of prey selectivity [22]. Despite the early usage and administration of large doses of *N. atra* antivenom in envenomed patients, severe wound necrosis or chronic necrotic ulceration causing extensive local tissue injuries are commonly reported [9,10,11]. Although subsequent wound infection due to heavy bacterial load introduced by the fangs [23,24] might contribute to this clinical dilemma, there is the possibility of a lack of efficacy of the specific antivenom against the myotoxic effect of the venom.

As *N. atra* venom significantly inhibited responses to KCl in the indirectly stimulated chick biventer experiments, the presence of myotoxic activity in the venom was further examined in the directly stimulated chick biventer preparation. *N. atra* venom abolished direct twitches and induced an increase in baseline tension indicative of myotoxicity [14,15]. Prior addition of *N. atra* antivenom delayed, but did not prevent, inhibition of direct twitches, but abolished the increase in baseline tension. However, the inability of *N. atra* antivenom to fully prevent myotoxicity may not indicate a lack of full efficacy of the antivenom. Given that the myotoxic effects were studied at 10× the concentration at which the neurotoxic effects were studied (i.e., 30 μg/mL compared to 3 μg/mL), a ratio which is in line with many of our previous neurotoxic/myotoxic studies [25,26,27], it is likely that increasing the antivenom concentration, e.g., at least double the manufacturer’s recommended amount, may full prevent the myotoxic effects. Indeed, we needed to add 2× the manufacturer’s recommendation to abolish venom neurotoxicity. However, we limited the maximum amount of antivenom used in the myotoxicity study given that excessive amounts of antivenom can alter the osmolarity of the physiological salt solution in the organ bath and affect tissue viability. The Australian polyvalent snake antivenom and the two Chinese monovalent viper antivenoms failed to significantly inhibit either the decrease in twitch height or increase in baseline tension.

*N. atra* antivenom also neutralized the potent cytotoxic effects of *N. atra* venom in L6 rat skeletal muscle cells. It is worth noting that the antivenom was protective against 30 μg/mL of venom in this assay, further supporting that the lack of full efficacy in the chick biventer myotoxic study was due to an insufficient concentration of antivenom. This problem did not occur in the cell assay given the much lower volumes used. The Australian polyvalent snake antivenom was protective at lower concentrations of venom in the cytotoxicity assay, whereas the two viper antivenoms had no significant protective effect. Although the venoms from these Chinese viper species (i.e., *G. brevicaudus* and *D. acutus*) can cause local tissue swelling and necrosis in envenomed humans, their venom proteomes and the relative abundance of major components are quite different to the Chinese elapid *N. atra* venom [3,5]. Therefore, it is not surprising that these antivenoms were unable to prevent the myotoxicity and cytotoxicity induced by *N. atra* venom. Our results strongly suggest that these viper antivenoms are unlikely to neutralize the effects of venom in patients envenomed by *N. atra*. Interestingly, the Australian polyvalent snake antivenom, which is raised against the venoms from five species of highly venomous terrestrial Australian elapids, failed to prevent *N. atra* venom induced myotoxicity in vitro while showing capability of fully preventing and even partially reversing *N. atra* venom induced neurotoxicity and cytotoxicity. This divergence has not been previously reported and could be explained by further venomic comparison studies between the species in the future.

## 5. Conclusions

In summary, we have, for the first time, examined the in vitro neurotoxic, myotoxic, and cytotoxic effects of *N. atra* venom and the ability of specific Chinese *N. atra* monovalent antivenom, non-specific Australian polyvalent snake antivenom, and Chinese *G. brevicaudus* monovalent antivenom and Chinese *D. acutus* monovalent antivenom to neutralize these effects. Our studies indicate that Chinese *N. atra* venom causes potent in vitro neurotoxicity, myotoxicity, and cytotoxicity, which is, largely, neutralized by *N. atra* antivenom. While the Australian polyvalent antivenom was equally efficacious against the neurotoxic effects, indicating the presence of similar antigenic neurotoxins, it was ineffective against the myotoxicity and only partially protective against the cytotoxic effects. The Chinese viper antivenoms were ineffective and do not appear to display any cross-reactivity against the myotoxic and cytotoxic components of *N. atra* venom.
